# Genome-Wide Analysis and Characterization of Aux/IAA Family Genes in *Brassica rapa*

**DOI:** 10.1371/journal.pone.0151522

**Published:** 2016-04-06

**Authors:** Parameswari Paul, Vignesh Dhandapani, Jana Jeevan Rameneni, Xiaonan Li, Ganesan Sivanandhan, Su Ryun Choi, Wenxing Pang, Subin Im, Yong Pyo Lim

**Affiliations:** Molecular Genetics and Genomics Laboratory, Department of Horticulture, Chungnam National University, Daejeon, 305764, South Korea; Huazhong university of Science and Technology, CHINA

## Abstract

Auxins are the key players in plant growth development involving leaf formation, phototropism, root, fruit and embryo development. Auxin/Indole-3-Acetic Acid (Aux/IAA) are early auxin response genes noted as transcriptional repressors in plant auxin signaling. However, many studies focus on Aux/ARF gene families and much less is known about the Aux/IAA gene family in *Brassica rapa* (*B*. *rapa*). Here we performed a comprehensive genome-wide analysis and identified 55 Aux/IAA genes in *B*. *rapa* using four conserved motifs of Aux/IAA family (PF02309). Chromosomal mapping of the *B*. *rapa* Aux/IAA (*BrIAA*) genes facilitated understanding cluster rearrangement of the crucifer building blocks in the genome. Phylogenetic analysis of *BrIAA* with *Arabidopsis thaliana*, *Oryza sativa* and *Zea mays* identified 51 sister pairs including 15 same species (*BrIAA*—*BrIAA*) and 36 cross species (*BrIAA*—*AtIAA*) *IAA* genes. Among the 55 *BrIAA* genes, expression of 43 and 45 genes were verified using Genebank *B*. *rapa* ESTs and in home developed microarray data from mature leaves of Chiifu and RcBr lines. Despite their huge morphological difference, tissue specific expression analysis of *BrIAA* genes between the parental lines Chiifu and RcBr showed that the genes followed a similar pattern of expression during leaf development and a different pattern during bud, flower and siliqua development stages. The response of the *BrIAA* genes to abiotic and auxin stress at different time intervals revealed their involvement in stress response. Single Nucleotide Polymorphisms between *IAA* genes of reference genome Chiifu and RcBr were focused and identified. Our study examines the scope of conservation and divergence of Aux/IAA genes and their structures in *B*. *rapa*. Analyzing the expression and structural variation between two parental lines will significantly contribute to functional genomics of *Brassica* crops and we belive our study would provide a foundation in understanding the Aux/IAA genes in *B*. *rapa*.

## Introduction

Auxin is the most important hormone involved in many plant organ development processes like root, flower, leaf and fruit, and regulates plant responses including phototropism, gravitropism, apical dominance and cell elongation [1, 2]. Auxin/indole-3-acetic acid (Aux/IAA) hormones are primary responsive auxin genes that are short-lived and localize in nucleus, conversely some Aux/IAA proteins are long-lived and not degraded easily. The Aux/IAA proteins are generally conserved with four characteristic motifs referred to as domain-I, -II, -III and -IV, however proteins lacking one or two of these domains were also included in this gene family [1]. Domain-I contains a leucine rich repeat motif represented by “LxLxL” and functions as a repression domain that interacts with TOPLESS (*TPL*) co-repressor [3]. Domain-II is highly conserved playing a major role in protein stability and it’s required for auxin-regulated signaling by interacting with a component of transport inhibitor response 1 (*TIR1*) [4]. Mutations occurring in this domain affect the interaction and results in low auxin response. Domain-III and -IV are involved in Aux/IAA and/or ARF proteins homo- and hetrodimerization [1]. Aux/IAA proteins bind to the associate ARF proteins by the domains III/IV and repress the ARF activity. Aux/IAA proteins are ubiquitinated through auxin induced SCF^TIR1^ complex and degraded by 26S proteasome in active condition [4]. Studying the auxin regulator activity is highly complex due to the large number of Aux/IAA gene families, variations, expression patterns, auxin mediated transcriptional and posttranscriptional regulations.

To understand the molecular mechanism of Aux/IAA genes, they were isolated and studied in different plant species like soybean, pea, *Arabidopsis thaliana*, *Populus trichocarpa* and Rice [5–8]. In *A*. *thaliana* the Aux/IAA genes were identified to mediate light response. In tomato the genes were noted as active repressors of auxin dependant gene transcription. Recently in cucumber and citrus their association with auxin induction and development of citrus organs have been analyzed and reported. It was also found that Aux/IAA proteins influence root initiation, vascular and organ development, apical dominance, flower and fruit growth of plant system. Other than their involvement in plant development their role during early phase of *Sinorhizobium meliloti* infection were also analyzed in *Medicaga truncatula*. Despite the knowledge of Aux/IAA gene family, in plants like chickpea and soybean they have just been identified and many questions about their function remained unknown. Information of *Aux/IAA* genes in vegetable crops is very limited and uncharacterized especially in *Brassica* genome. Recent publication of the *Brassica rapa* whole genome sequence provided a quantum leap in understanding the genome structure and facilitated the characterization of many genes [9]. Studying the role of Aux/IAA genes in *B*. *rapa*, a model organism for the *Brassica* species, could provide better understanding about the functional attributes of this gene family in *Brassicas* and also stimulate further researches in related organisms. Here we aimed the genome wide identification and organization of Aux/IAA genes, prediction of the related proteins domains and their architechture and furthermore, to identify the single nucleotide polymorphisms (SNPs) between the Aux/IAA genes of Chiifu and Rapid cycling *B*. *rapa* (RcBr) inbred lines. The identified putative candidate genes were utilized to trace the *A*. *thaliana* homologues genes, similar *B*. *rapa* ESTs from GenBank and expressed sequences from in-home developed chip sequences of Chiifu and RcBr inbred lines by bioinformatics using comparative analysis. Expression of multiple *B*. *rapa IAA* genes were checked by Semi quantitative real time PCR (semi qRT-PCR) analysis and an auxin treatment was carried out to analyze the expression pattern of the genes. The obtained would expedite future research to analyze in detail the functions of Aux/IAA genes in *Brassica*.

## Materials and Methods

### Isolation of *B*. *rapa Aux/IAA* family genes

All 41,019 genes of *B*. *rapa* were downloaded from BRAD database (http://Brassicadb.org/brad/). The alignments of Aux/IAA gene family under Pfam (http://pfam.sanger.ac.uk/) accession PF02309 in Stockholm format was used for a Hidden Morkov Model (HMM) search. The PF02309 based domains in *B*. *rapa* proteins were identified using HMMER software with E-value cut off 1.0. Later a Pfam batch search was performed to the filtered *B*. *rapa* genes obtained from HMM search to confirm gene family. Then SMART (http://smart.embl-heidelberg.de/) and InterProScan (http://www.ebi.ac.uk/Tools/pfa/iprscan/) web servers were used to examine the conserved domains from identified *B*. *rapa* genes. Finally the candidate *B*. *rapa* Aux/IAA (*BrIAA*) genes were compared to UniProt Knowledgebase (UniProtKB—http://www.uniprot.org/help/uniprotkb) to validate with their homologues in other species.

### Motif discovery and Phylogenetic of *B*. *rapa Aux/IAA* proteins

The conserved motifs of *B*. *rapa*, *A*. *thaliana*, *O*. *sativa* and *Z*. *mays* Aux/IAA genes were identified using MEME web server (http://meme.nbcr.net). Motifs’ satisfying the following criteria were only considered: (i) the occurrences of a single motif among the sequences allowed to zero or one per sequence; (ii) Optimum width of each motif limits between 5 to 300; (iii) Five maximum number of motifs admitted to search. Multiple sequence alignment was performed by downloading the Aux/IAA genes of *A*. *thaliana*, *O*. *sativa* and *Z*. *mays* and aligning with *BrIAA* genes using ClustalX algorithm. Phylogenetic tree was generated using neighbor-joining method with 1000 bootstrap test by MEGA 5.05 software.

### Mapping *BrIAA* in the genome of *B*. *rapa*

Chromosomal distributions of *BrIAA* were determined by comparing the identified sequence of the *BrIAA* to whole genome sequence of *B*. *rapa* downloaded from BRAD database. The comparison was done by BLAT software (http://genome.ucsc.edu/cgi-bin/hgBlat). The structure of each gene was retrieved from BRAD database GFF file using our own perl script (The scripts are available on request). Resulted genomic sequence structure was examined by Gene Structure Display Server (http://gsds.cbi.pku.edu.cn/).

### Comparative analysis and Expression profiling of *BrIAA*

The recent version of *A*. *thaliana* gene models (41,671) was downloaded from TAIR database (http://arabidopsis.org/) for a synteny analysis between genomes since *A*. *thaliana* is close relative of *B*. *rapa*. We performed megaBLAST and annotated similar genes along with the genomic block information by combining the similar *A*. *thaliana* genes and genome location in *B*. *rapa*. Additionally over 200,000 *B*. *rapa* ESTs were downloaded from NCBI GenBank EST database and assembled to find the unigenes by CAP3 software (http://pbil.univ-lyon1.fr/cap3.php) after removing repeats and low complexity sequence by RepeatMasker software (http://www.repeatmasker.org/) following similar methods of our previous publication [10]. Further another megaBLAST was performed to identify the similar unigenes for *B*. *rapa* Aux/IAA genes. Go annotations were then carried out for the identified unigenes using blast2go utility.

### RNA isolation, IAA treatment and RT-PCR

Total RNA was extracted from 20-day-old leaf, bud, flower and siliqua samples of Chiifu, RcBr lines and semi quantitative RT-PCR analysis was performed using Aux/IAA gene-specific primer pairs listed in S1 Table. We used specific primer “BrActin” for *B*. *rapa* actin gene sequence as a control. One μg total RNA was reverse transcribed to first strand cDNAs using oligo (dT) 20 primer and ReverTra Ace reverse transcriptase (ToYoBo co.). The synthesis cDNAs were used as templates for PCR amplification as followed: 94°C for 5 min, 25–30 cycles of 94°C for 30s, 52°C for 30s, 72°C for 1 min, and final extension of 72°C for 7 min. The PCR products were then electrophoreses on a 1.2–1.5% agarose gel.

For IAA treatment *B*. *rapa* seeds were surface sterilized, rinsed in sterile double distilled water and sown in Magenta vessels containing 50 ml of MS basal medium with 3% (w/v) sucrose and 0.8% (w/v) agar, pH 5.8. Plants were grown under light/dark condition with 24±26°C. After 10 days of culture, the seedlings (approx. 12 cm length) were transferred to MS liquid medium containing constant concentration of IAA (10 μM) and were collected at various time intervals (0, 24, 48, 72, and 96 h). Samples were put in liquid nitrogen and immediately stored in -80°C freezer. Now a semi quantitative RT-PCR was carried out using total RNA from leaf explants of IAA treated *B*. *rapa* and control plants by Trizol method. Semi-quantitative reverse transcription-polymerase chain reaction (RT-PCR) analysis was performed using the Aux gene-specific primer pairs (S1 Table). We used the specific primer ‘BraACTIN’ for the *B*. *rapa* actin gene sequences as a control again. One microgram of total RNA was reverse transcribed to the first-strand cDNAs by using an oligo (dT) 20 primer and RevertAid Reverse Transcriptase (Thermo Scientific). The synthesis cDNAs were used as templates for PCR amplification as follows: 94°C for 5 min; 25–30 cycles of 94°C for 30s, 60°C for 30s, and 72°C for 1 min; and a final extension at 72°C for 7 min. The PCR products were then electrophoresed on a 1.0% agarose gel.

Real Time PCR was performed to quantify the stress treated plants with IAA treated *B*. *rapa* with a comparision to non-treated plants in order to evaluate the aux gene expression pattern. The synthesized cDNA (RevertAid Reverse Transcriptase) was diluted to 1:5 in DEPC water and subjected to qRT PCR analysis using SYBR Green Supermix in Crmatos instrument. BraActin gene was used as an internal control to estimate the relative transcript level of the gene tested. The conditions of the qRT-PCR are as follows 10 min at 95°C, followed by 40 cycles at 95°C for 15 s, 52°C for 20 s, and 72°C for 15 s. Relative fold differences were calculated based on the comparative Ct method (2^-ΔΔCt^). Three replicates were performed for each gene for qRT PCR analysis and heat map representation was illustrated using Ct value in online tool (CIMminer) (http://discover.nci.nih.gov/cimminer/home.do).

### Microarray analysis of Aux/IAA genes in *B*. *rapa* Chiifu and RcBr lines

Our in-home developed and unpublished microarray information of Chiifu and RcBr inbred lines of *B*. *rapa* having wide morphological diversity, and was chosen to analyze the expression pattern of *BrIAA* genes. The microarray was prepared by isolating total RNA from mature healthy leaves of Chiifu and RcBr (inbred lines of *B*. *rapa*) and subjected to synthesis of Cy3-labeled target DNA fragments containing *B*. *rapa* 47,548 unigenes, manufactured by NimbleGen, Inc. (http://www.nimblegen.com/). To assess the reproducibility of the microarray analysis, we repeated the experiment twice using independently prepared total RNAs. The data were normalized and processed with cubic spline normalization using quantiles to adjust signal variations between chips and Robust Multi-Chip Analysis (RMA) using a median polish algorithm implemented in NimbleScan (Workman et al., 2002; Irizarry et al., 2003). The *BrIAA* genes were compared to the microarray chip sequences using locally installed megaBLAST program, sequences with E-value cut-off 0.0001 and >98% similarity were considered as expressed sequences. Redundant sequences were removed and the gene family was confirmed using BLASTx program. Fold values were also calculated for expressed *BrIAA* genes.

We also utilized the cold, salt and drought treated KBGP-24K microarray to verify the expression of the identified genes [11].

### SNP annotation between the genes of *B*. *rapa* Chiifu and RcBr lines

Whole genome re-sequences of *B*. *rapa* parental line RcBr using Illumina GAII next generation sequencer with 30x coverage. After filtering and trimming low quality reads we achieved over 27x coverage. Draft genome *B*. *rapa* Chiifu line was used as reference genome to assembled RcBr reads with Bowtie2 software (http://bowtie-bio.sourceforge.net/index.shtml). SNPs and other variations were annotated by SAM and BCF tools following the provided manual (http://samtools.sourceforge.net/). Identified *B*. *rapa* Aux/IAA genes were mapped to the RcBr genome and SNPs were mined by our own Perl script and confirmed with BLAT software.

## Results

### Identification and characterization of Aux/IAA family genes in *B*. *rapa*

HMM based search for Aux/IAA gene family in *B*. *rapa* with Pfam accession PF02309 resulted in identification of 88 genes with Aux/IAA domain. After a careful verification 23 genes identified with B3 and ARF domains confirming auxin response factor gene family were removed. The resulted sequences were then searched again using Pfam batch search and 55 B. *rapa* genes with confidant Aux/IAA domain were confirmed as representatives of *BrIAA* gene family after a manual curation. All identified genes were searched against UniProt database using megaBLAST to further confirm the identification and found most of the top hits were matching Aux/IAA proteins from *A*. *lyrata* and *A*. *thaliana* genomes. The domains of the candidate genes were examined by SMART and InterProScan web servers. Forty five of the total genes were conserved with only one Aux/IAA domain, the remaining four genes consisted two Aux/IAA domain leaving the rest five with both Aux/IAA and non auxin domains like PB1, Psb28 and AP2 domains. Identified genes were named from *BrIAA*1 to *BrIAA*55 based on their domain conservation (Table 1).

**Table 1:** The index of Aux/IAA genes in *B*. *rapa*.

**Gene**	Chr	BRAD ID	Exons & Introns	Length	Unigene ID	At ID	Pfam domain
*BrIAA1*	A09	Bra032520	5&4	1913	Contig5385	AT1G04250	Aux/IAA
*BrIAA2*	A08	Bra030560	5&4	1577	Contig9475	AT1G04250	Aux/IAA
*BrIAA3*	A10	Bra015298	5&4	1391	Contig1004	AT1G04250	Aux/IAA
*BrIAA4*	A10	Bra015297	3&2	723	FY418515	AT1G04240	Aus/IAA
*BrIAA5*	A10	Bra015326	5&4	1161	EX101336	AT1G04550	Aux/IAA
*BrIAA6*	A09	Bra032503	7&6	2360	EX106196	AT1G04550	Aux/IAA
*BrIAA7*	A09	Bra026755	3&2	655	L37504	AT1G15580	Aux/IAA
*BrIAA8*	A10	Bra015289	4&3	1219	Contig7455	AT1G04100	Aux/IAA
*BrIAA9*	A08	Bra014303	5&4	1409	EX109423	AT1G51950	Aux/IAA
*BrIAA10*	A06	Bra018938	5&4	1312	EX094947	AT1G51950	Aux/IAA
*BrIAA11*	A06	Bra018993	3&2	1038	GR720370	AT1G52830	Aux/IAA
*BrIAA12*	A07	Bra035185	4&3	904	EX015600	AT1G80390	Aux/IAA
*BrIAA13*	A03	Bra001900	5&4	1848	Contig3476	AT3G23050	Aux/IAA
*BrIAA14*	A05	Bra033886	5&4	1929	Contig12621	AT3G23050	Aux/IAA
*BrIAA15*	A01	Bra040122	4&3	1445	FY419128	AT3G04730	Aux/IAA
*BrIAA16*	Scaffold000203	Bra040398	5&4	1537	Contig1782	AT3G04730	Aux/IAA
*BrIAA17*	A05	Bra022164	5&4	1469	Contig8735	AT3G16500	Aux/IAA
*BrIAA18*	A01	Bra021184	5&4	1381	EX025736	AT3G16500	Aux/IAA
*BrIAA19*	A01	Bra021117	3&2	1158	EX089451	AT3G15540	Aux/IAA
*BrIAA20*	A03	Bra001598	3&2	1090	EX128302	AT3G15540	Aux/IAA
*BrIAA21*	A05	Bra027232	3&2	1243	Contig5848	AT3G15540	Aux/IAA
*BrIAA22*	A03	Bra001634	5&4	1299	Contig5860	AT3G16500	Aux/IAA
*BrIAA23*	A04	Bra030219	5&4	1119	Contig18210	AT2G22670	Aux/IAA
*BrIAA24*	A03	Bra022934	4&3	1231	Contig1521	AT2G33310	Aux/IAA
*BrIAA25*	A05	Bra005508	4&3	1116	EX101243	AT2G33310	Aux/IAA
*BrIAA26*	A04	Bra040476	3&2	862	NA	AT2G46990	Aux/IAA
*BrIAA27*	A07	Bra003484	3&2	1005	NA	AT3G62100	Aux/IAA
*BrIAA28*	A09	Bra007661	3&2	930	EE524500	AT3G62100	Aux/IAA
*BrIAA29*	A04	Bra014411	3&2	890	Contig15454	AT3G62100	Aux/IAA
*BrIAA30*	A01	Bra021257	3&2	401	NA	NA	Aux/IAA
*BrIAA31*	A06	Bra024898	3&2	675	NA	AT2G01200	Aux/IAA
*BrIAA32*	A02	Bra020243	3&2	649	NA	AT5G60450	Aux/IAA
*BrIAA33*	A09	Bra036557	4&3	1128	EX100611	AT5G25890	Aux/IAA
*BrIAA34*	A08	Bra039855	5&4	1883	EX110195	AT4G14550	Aux/IAA
*BrIAA35*	A08	Bra039732	2&1	599	EX111917	AT4G14560	Aux/IAA
*BrIAA36*	A01	Bra011082	5&4	1297	Contig10439	AT4G29080	Aux/IAA
*BrIAA37*	A01	Bra011045	5&4	1395	NA	AT4G28640	Aux/IAA
*BrIAA38*	A08	Bra010338	4&3	823	Contig10439	AT4G29080	Aux/IAA
*BrIAA39*	A01	Bra011332	4&3	1037	NA	AT4G32280	Aux/IAA
*BrIAA40*	A03	Bra023958	4&3	1152	NA	AT4G32280	Aux/IAA
*BrIAA41*	A09	Bra027504	3&2	755	EX050560	AT5G43700	Aux/IAA
*BrIAA42*	A10	Bra002729	2&1	941	NA	AT5G57420	Aux/IAA
*BrIAA43*	A09	Bra037824	5&4	909	FY072042	AT5G65670	Aux/IAA
*BrIAA44*	A02	Bra031850	5&4	1204	EX096729	AT5G65670	Aux/IAA
*BrIAA45*	A06	Bra024406	5&4	1151	Contig17855	AT5G65670	Aux/IAA
*BrIAA46*	A09	Bra031069	5&4	1878	Contig3427	AT1G19220	Aux/IAA, AP2
*BrIAA47*	A09	Bra032521	3&2	734	FY418515	AT1G04240	Aux/IAA, Aux/IAA
*BrIAA48*	A05	Bra033890	2&1	607	Contig6739	AT3G23030	Aux/IAA, Aux/IAA
*BrIAA49*	A03	Bra001899	2&1	603	FY070711	AT3G23030	Aux/IAA, Aux/IAA
*BrIAA50*	A01	Bra023771	2&1	600	EX111917	AT3G23030	Aux/IAA, Aux/IAA
*BrIAA51*	A06	Bra026166	4&3	1280	NA	AT1G15050	Aux/IAA, EIIBC-GUT_N, Rhabdo_NV
*BrIAA52*	A03	Bra001670	5&4	792	NA	AT3G17600	Aux/IAA, PB1
*BrIAA53*	A06	Bra009867	4&3	1131	EX099226	AT5G25890	Aux/IAA, PB1
*BrIAA54*	A03	Bra024187	5&4	2750	Contig13041	AT4G28640	Aux/IAA, Psb28
*BrIAA55*	A05	Bra022224	4&3	738	NA	AT3G17600	Aux/IAA, Ribosomal_L30,PB1

Chr—chromosome; At ID—*A*. *thaliana* ID; NA—not available.

**Fig 1:**
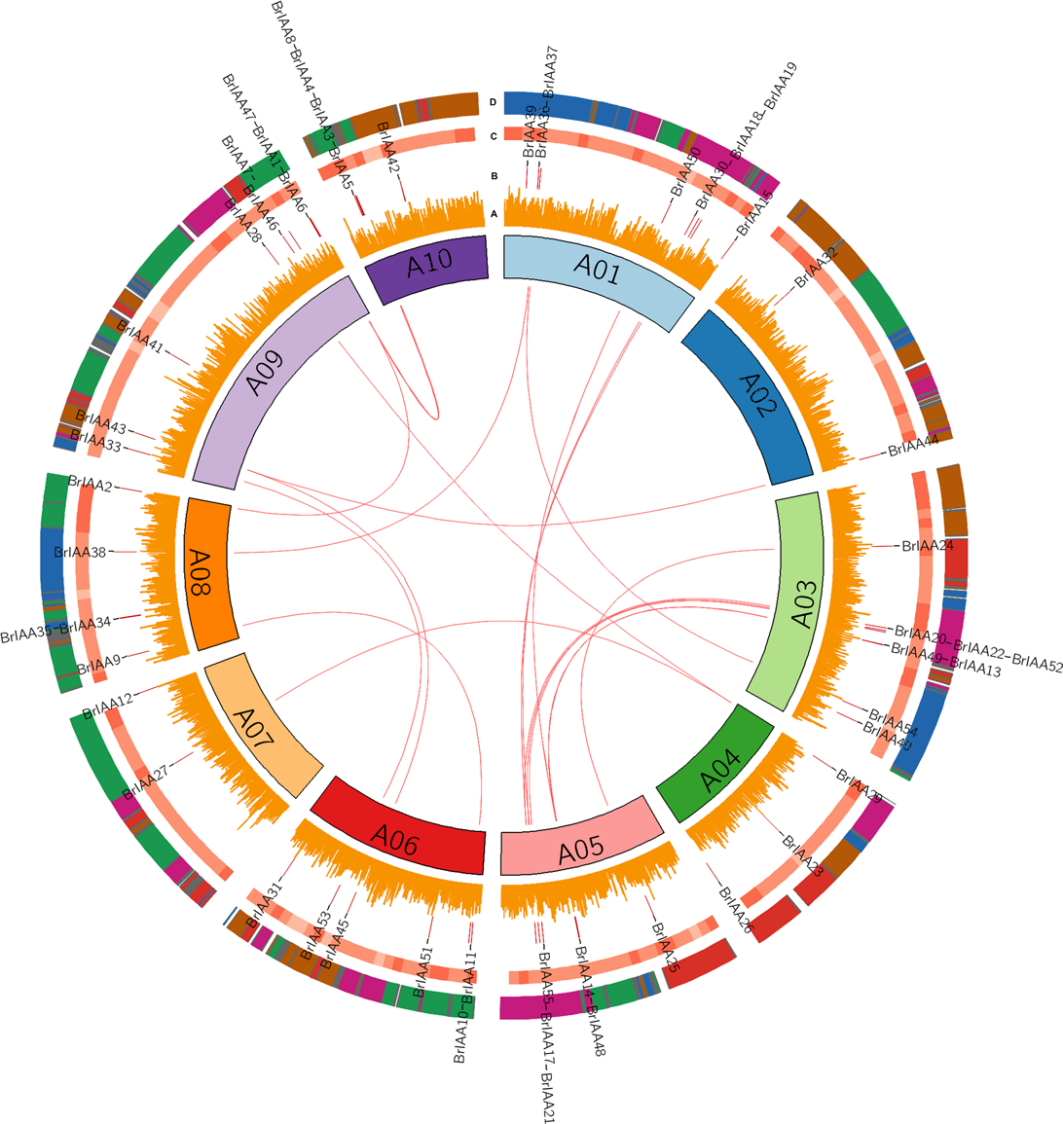
Chromosome positions of *BrIAA* genes. From inner to outer ring A) Single Nucleotide Polymorphisms identified between the RcBr and Chiifu line. B) Physical positions of the Aux/IAA genes in the genome. C) GC % per 1Mb is illustrated in 3 different shades lesser GC% lighter the color. D) Shows the homologues crucifier building blocks of each gene and their clusters of the *BrIAA* genes. The colors representing the blocks are described as follows: green- A, B, C, D, E; red- G, H, I, J, K; pink- F, L, M, N; blue-O, P, T, U; brown- Q, R, S, V, W, X and white- nil.

*BrIAA* genes were mapped to all chromosomes of *B*. *rapa* except *BrIAA*16, which was mapped to scaffold000203 from BRAD database. Maximum number of genes *i*.*e* 8, 8 and 9 genes was localized on A01, A03 and A09 chromosome (Fig 1). Genome blocks of each gene were identified by comparing with the *A*. *thaliana* genome blocks [12]. Totally 50 genes were mapped to 14 genome blocks with 14 and 11 number of *BrIAA* genes in F and A blocks, respectively. *BrIAA15*, *BrIAA38 and BrIAA53* genes’ blocks were unknown, however, it was mapped in their respective chromosome without block information in Fig 1. Intronic and exonic regions were identified for each *BrIAA* gene by comparing the genomic sequences to genes and validated by *B*. *rapa* gene gff file. Most number of genes having 3 to 5 exons was conserved with single domain Aux/IAA. However, four genes *BrIAA*47, *BrIAA*48, *BrIAA*49 and *BrIAA*50 found with two IAA domains had lower number of exons and smaller sequence length than the average *BrIAA* genes (Table 1).

### Phylogeny and motif distribution of *B*. *rapa* Aux/IAA proteins

**Fig 2:**
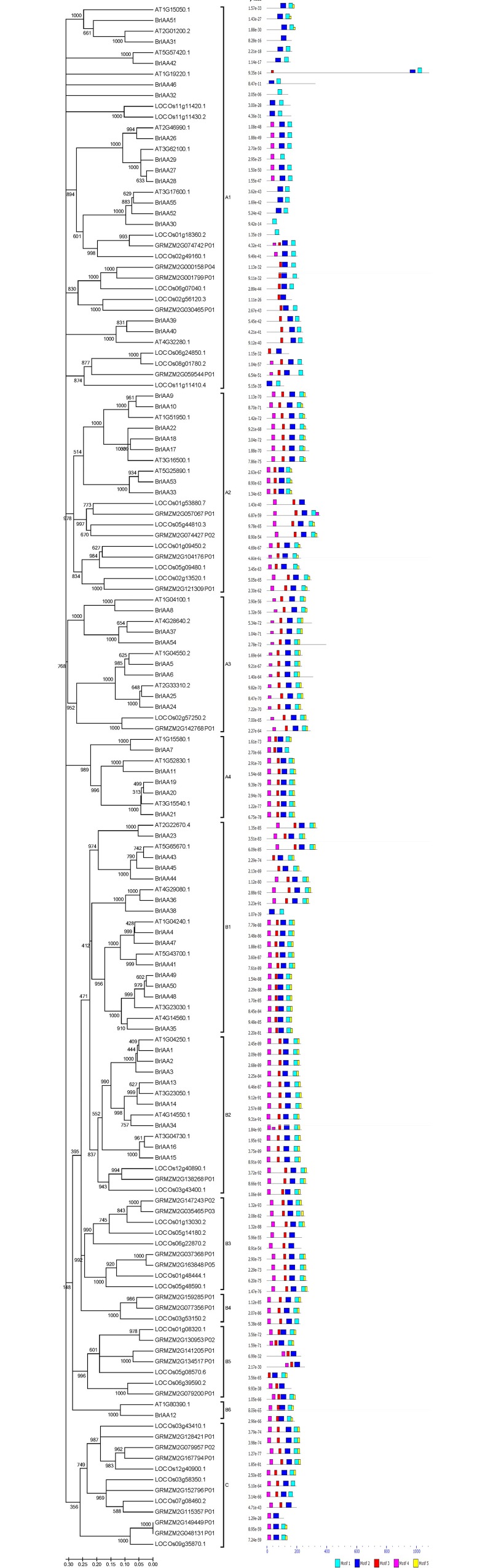
Phylogenetics analysis of full length Aux/IAA protein sequences from *A*. *thaliana*, *B*. *rapa*, *O*. *sativa* and *Z*. *mays* using neighbor-joining method. Motifs for each protein were predicted by MEME web server. Group (A, B and C) and their subgroups were distinguished by phylogeny conservation of Aux/IAA genes.

In order to examine the phylogenetic relationship among *B*. *rapa*, *A*. *thaliana*, *O*. *sativa and Z*. *mays* Aux/IAA genes, we aligned the amino acid sequences of 144 genes by Clustalx and generated a rooted phylogenetic tree by MEGA software. The generated tree was divided into three groups, namely A, B and C based on their phylogeny. Both the phylogenetic tree and motif conservation are well matched to previously studied plants *A*. *thaliana*, Solanaceae species (potato and tomato) and *Vitis vinifera* [13, 14]. Later A and B groups were divided into 4 (A1-A4) and 6 (B1-B6) subgroups for better understanding (Fig 2). Among A subgroups 14 monocotyledon (*O*. *sativa* and *Z*. *mays*) and 22 dicotyledon (*A*. *thaliana* and *B*. *rapa*) IAA genes belong to group A1. Another subgroup A2 contained 9 monocot and 10 dicot IAA genes. However subgroup A3 and B2 had larger number of IAA genes from dicots than monocots. Also A4, B1 and B6 groups were found with only *A*. *thaliana* and *B*. *rapa* IAA genes. Similarly subgroups B3, B4, B5 and group C contained only *O*. *sativa* and *Z*. *mays* IAA proteins.

In the phylogentic tree, a total of 51 sister pairs were found, that includes 15 from same species (*BrIAA*—*BrIAA*) IAA genes and 36 from cross species (*BrIAA*—*AtIAA*) IAA genes. Total 6 pairs were found for *B*. *rapa* IAA genes (*BrIAA*—*BrIAA*) among same species sister pairs. Also 2 and 7 numbers of pairs were found for *O*. *sativa* (*OsIAA*—*OsIAA*) and *Z*. *mays* (*ZmIAA*—*ZmIAA*) respectively. Between 30 *A*. *thaliana* IAA genes, there is no single sister pair found in the whole tree. However 23 pairs were attained between *B*. *rapa* and *A*. *thaliana* IAA genes (*BrIAA*—AtIAA) as cross species sister pair. Similarly 13 pairs were identified between *O*. *sativa* and Z. *mays* IAA genes (*OsIAA*—*ZmIAA*). Subgroup A1 comprised of all five types of sister pairs (*BrIAA*—*BrIAA*, *OsIAA*—*OsIAA*, *ZmIAA*—*ZmIAA*, *BrIAA*—*AtIAA*, *OsIA*A—*ZmIAA*) one or more than one time (Fig 2).

The possible motifs were searched for all 144 IAA proteins incuding 30 *A*. *thaliana*, 55 *B*. *rapa*, 31 *O*. *sativa* and 28 *Z*. *mays* by Multiple Expectation maximization for Motif Elicitation (MEME) web server (http://meme-suite.org/tools/meme). Predicted five motifs were designated to four conserved domains of Aux/IAA proteins. Domain-I, -II and -III were represented by motif-4, -3 and -2, though longest domain-IV was constituted by motif-1 and -5 (Fig 2; S1 Fig). Totally four and fourteen IAA proteins were conserved with only single and double motifs respectively. Among single motif containing proteins, three were *B*. *rapa* genes namely *BrIAA30*, *BrIAA31 and BrIAA32*, and remaining one was *O*. *sativa* protein LOCOs01g18360.2 conserved with only motif-2 (Fig 2). The number of sites for each motifs and their e-value were presented in S1 Fig

### Homologues *A*. *thaliana* genes and *B*. *rapa* ESTs

Identifying homologues *B*. *rapa* genes in model organism *A*. *thaliana* is mandatory since both the genome belongs to Brassicacea family. Discovered *BrIAA* genes were compared to all gene coding sequences of *A*. *thaliana* and identified 31 unique auxin gene models which were highly homolog to 54 *BrIAA* genes except for *BrIAA*30 gene, however it’s well conserved with single Aux/IAA domain. Among the 31 *A*. *thaliana* Aux/IAA genes, 14 and 17 genes have single and two or more than two copies of genes in *B*. *rapa* genome (Table 1).

Publically available *B*. *rapa* ESTs from NCBI were downloaded and assembled consequencing 46,954 unigenes with 19,234 contigs and 27,720 singlets. We obtained 43 *BrIAA* genes matching 19 contigs and 24 singlets by megaBLAST analysis with over 90% identity and 1e-100 e-value. *BrIAA*36 and *BrIAA*38 were highly matching to Contig10439, likewise EX111917 and FY418515 also matches to two *BrIAA* genes with higher similarity (Table 1). The *BrIAA*30 gene which was non-similar to *A*. *thaliana* was also not found in *B*. *rapa* unigenes. Assembled unigenes are accessible at http://brassest.cnu.ac.kr/unigene/Brunijan2013.fasta. *BrIAA* corresponding unigenes were compared to non-redundant database by BLASTx and verified with auxin homologues from different plant species. Gene ontology was carried out for all *BrIAA* unigenes using blast2go utility and their functions were annotated. As biological process, maximum 38 unigenes were response to endogenous stimulus in *B*. *rapa*. Additionally 35, 14, 11, and 9 *BrIAA* unigenes were response to signal transduction, abiotic stimulus, tropism and stress, respectively. In molecular level all the *BrIAA* unigenes were recognized with sequence-specific DNA and protein binding activity (S2 Fig).

### Aux treatment and Quantification by qRT PCR

**Fig 3:**
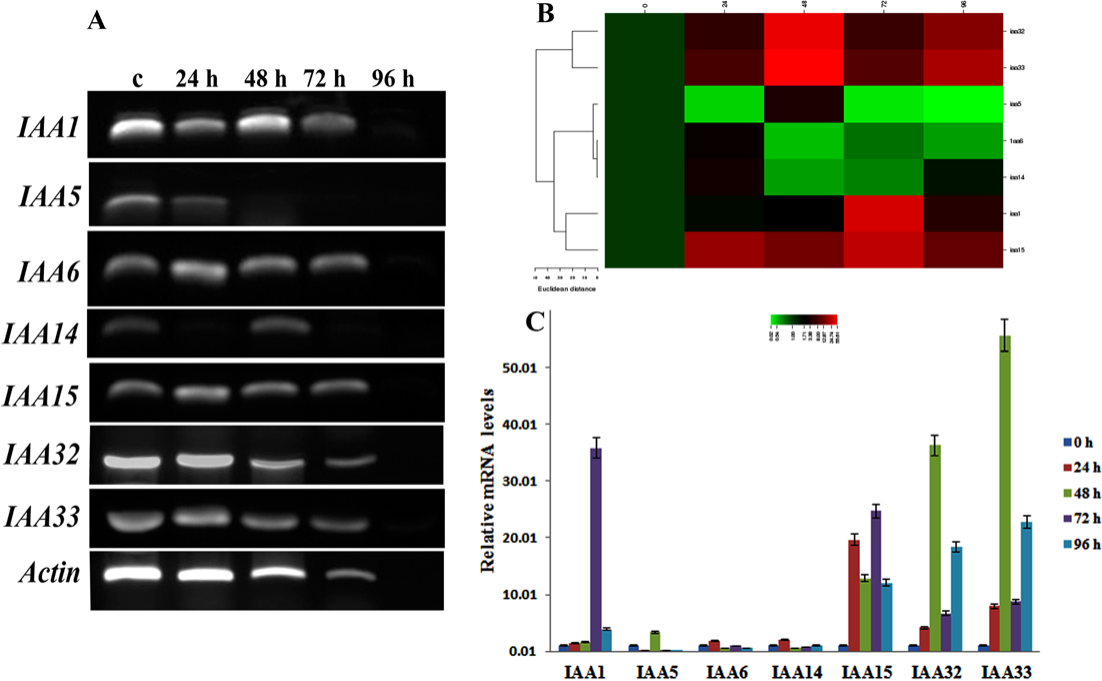
Response of *BrIAA* to Auxin treatment over a time course. A) Expression pattern of *BrIAA* genes to auxin treatment at different time points. B) Relative fold changes of the *BrIAA* genes in form of heatmaps. C) Transcript level of the genes during auxin treatment.

The Aux/IAA genes are highly induced by auxin, to verify this we exposed few of the *BrIAA* genes to Auxin treatment at different time course and validated their expression using sqRT PCR. Each *BrIAA* gene exhibited different expression pattern upon the treatment. In general the genes showed higher expression on lower time exposure and lower expression on higher exposure time. All *BrIAA* genes IAA1, IAA6, IAA14, IAA15, IAA32 and IAA33 expression were upregulated on 48 h of treatment than other exposure time except IAA5 gene (Fig 3A). On 96 h treatment, all *BrIAA* were down regulated. Meanwhile, five aux genes such as IAA1, IAA6, IAA15, IAA32 and IAA33 were expressed on all the exposure time except 96 h.

Also we analyzed the transcripts level using RT-PCR. The transcript levels for most of the aux genes were upregulated by IAA treatment compared to non treated plants irrespective of exposure time. Despite the fact of higher transcript levels in IAA treatment, quantities of transcripts varied depending upon the exposure time which is evident from the data illustrated in Fig 3C. The transcript levels of IAA15 were recorded moderately as 19.66, 12.87, 24.74, and 12.07 at 24, 48, 72, and 96 h, respectively. In the case of IAA33, the transcript levels exhibited higher (55.61) at 48 h exposure time followed by IAA32 at the same exposure time. Aux gene, IAA1 presented the maximum transcript level (35.85) under 72 h exposure time. Low levels of transcripts were documented for IAA5, IAA6 and IAA14 (Fig 3C).

### Expression of *B*. *rapa* Aux/IAA genes in parental lines Chiifu and RcBr

**Fig 4:**
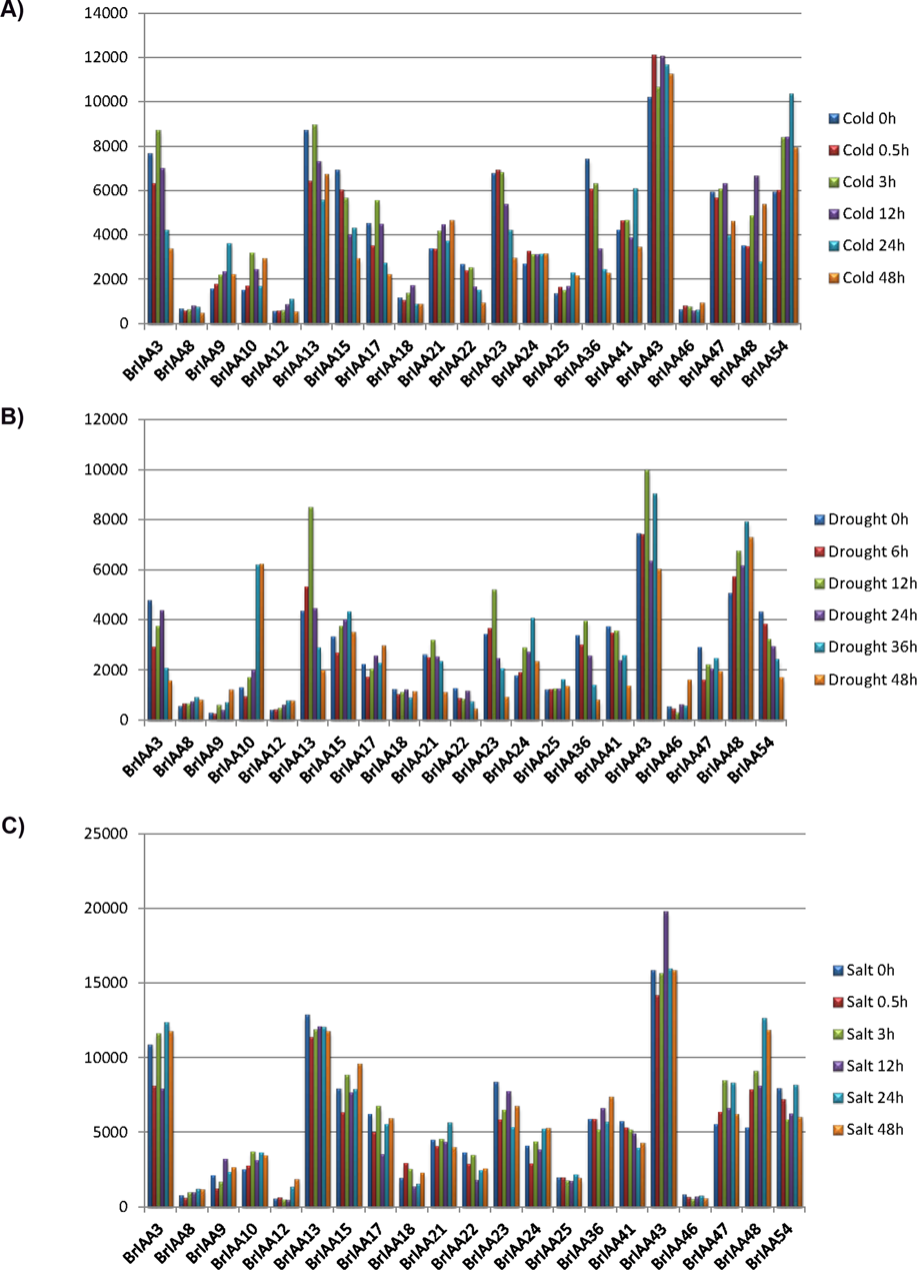
Stress response of *BrIAA* genes to cold, salt and drought treatments at various time points. Differentially expressed genes during A) cold B) drought C) salt treatments during various time. X axis represents the genes and Y axis represents the level of gene expression.

To understand the gene expression profiles in matured leafs of *B*. *rapa* Chiifu and RcBr inbred lines, we conducted a microarray experiment and obtained over 47,000 expressed genes with high similarity to *B*. *rapa* and/or *A*. *thaliana* genes. A total of 45 expressed genes were identified for *BrIAA* genes by megaBLAST algorithm with over 98% sequence coverage, >96% identity and 0.0 e-value. From the analysis, 13 genes were found to show over 1.5 fold change between parental lines Chiifu and RcBr (Table 2). A maximum of 8.11 fold change was attained for *BrIAA*51 gene which with high and low expression in RcBr and Chiifu lines respectively. Also 31 *BrIAA* genes were up-regulated and 14 were down regulated (up-regulated in Chiifu line) in RcBr. The maximum expression of 28,030 and 26,450 were obtained from Chiifu and RcBr for *BrIAA*54. The large portion of identified *BrIAA* genes expression level were over 1,000 in both Chiifu and RcBr inbred lines that indicates the involvement of IAA genes in leaf development of *B*. *rapa*. To further relate the involvement of the *BrIAA* to different abiotic stress like cold, salt and drought we performed a BLAST analysis with the stress treated KBGP-24K microarray and identified the differentially expressed genes. From the total *BrIAA* 21genes were found to be differently expressed to the different stress treatment. *BrIAA*43 showed a great expression change in all the cold, drought and salt treatments. During cold treatment *BrIAA*43 gene was upregulated in all time points but in drought and salt treatments the gene was down regulated initially and then upregulated (Fig 4).

**Table 2:** Expression ratio of *BrIAA* genes in Chiifu and RcBr inbred lines.

Gene	BrEST ID	Chiifu	RcBr	Chiifu/RcBr
*BrIAA1*	Brapa_ESTC022718	13,779	16,075	1.17
*BrIAA2*	Brapa_ESTC021484	11,472	12,689	1.11
*BrIAA3*	Brapa_ESTC005647	3,081	5,493	1.78
*BrIAA5*	Brapa_ESTC022012	6,832	7,052	1.03
*BrIAA6*	Brapa_ESTC039293	5,237	7,004	1.34
*BrIAA8*	Brapa_ESTC012149	7,384	7,368	-1.00
*BrIAA9*	Brapa_ESTC026941	21,024	17,159	-1.23
*BrIAA10*	Brapa_ESTC030931	24,912	16,467	-1.51
*BrIAA11*	Brapa_ESTC040927	612	782	1.28
*BrIAA12*	Brapa_ESTC015687	268	208	-1.28
*BrIAA13*	Brapa_ESTC003176	3,643	6,604	1.81
*BrIAA14*	Brapa_ESTC008873	11,767	13,829	1.18
*BrIAA15*	Brapa_ESTC027778	2,535	1,991	-1.27
*BrIAA16*	Brapa_ESTC013117	1,475	1,966	1.33
*BrIAA17*	Brapa_ESTC005402	5,284	3,833	-1.38
*BrIAA18*	Brapa_ESTC016467	5,512	6,117	1.11
*BrIAA19*	Brapa_ESTC020585	6,887	7,869	1.14
*BrIAA20*	Brapa_ESTC031156	30,595	19,821	-1.54
*BrIAA21*	Brapa_ESTC012631	7,285	6,507	-1.12
*BrIAA22*	Brapa_ESTC012609	9,687	10,986	1.13
*BrIAA23*	Brapa_ESTC002335	9,698	14,221	1.47
*BrIAA24*	Brapa_ESTC002137	4,345	7,230	1.66
*BrIAA25*	Brapa_ESTC033235	7,884	15,013	1.90
*BrIAA28*	Brapa_ESTC038343	5,347	6,126	1.15
*BrIAA32*	Brapa_ESTC032997	1,774	2,792	1.57
*BrIAA33*	Brapa_ESTC004462	2,747	6,611	2.41
*BrIAA34*	Brapa_ESTC022797	4,550	4,347	-1.05
*BrIAA36*	Brapa_ESTC026362	10,347	10,945	1.06
*BrIAA37*	Brapa_ESTC038600	7,294	9,785	1.34
*BrIAA38*	Brapa_ESTC030226	688	311	-2.21
*BrIAA39*	Brapa_ESTC044790	755	1,271	1.68
*BrIAA40*	Brapa_ESTC035154	472	1,126	2.38
*BrIAA41*	Brapa_ESTC000071	11,997	12,586	1.05
*BrIAA42*	Brapa_ESTC046479	30	43	1.42
*BrIAA43*	Brapa_ESTC046694	10,914	13,651	1.25
*BrIAA44*	Brapa_ESTC021498	15,980	21,485	1.34
*BrIAA45*	Brapa_ESTC012187	10,415	13,549	1.30
*BrIAA46*	Brapa_ESTC003213	6,158	7,195	1.17
*BrIAA47*	Brapa_ESTC039319	15,018	13,084	-1.15
*BrIAA48*	Brapa_ESTC002545	7,818	10,890	1.39
*BrIAA49*	Brapa_ESTC043297	11,884	11,511	-1.03
*BrIAA50*	Brapa_ESTC022957	16,659	15,448	-1.08
*BrIAA51*	Brapa_ESTC033550	250	2,023	8.11
*BrIAA53*	Brapa_ESTC021765	2,687	4,507	1.68
*BrIAA54*	Brapa_ESTC008162	28,030	26,450	-1.06

**Fig 5:**
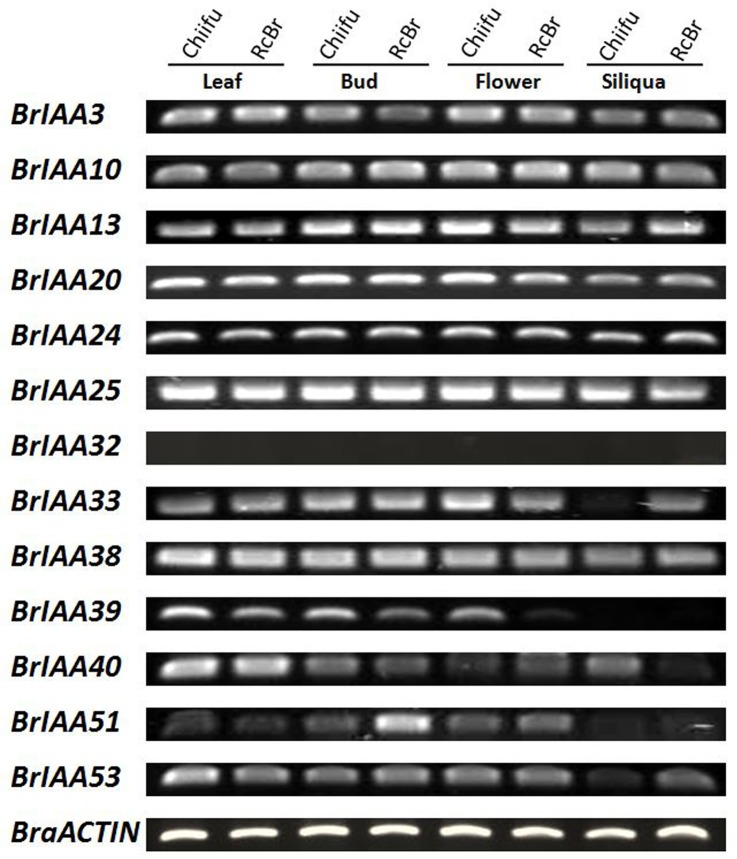
Expression of *BrIAA* genes in the different organs of ‘Chiifu’ and ‘RcBr’ lines were analyzed by semi qRT-PCR. *BrIAA32* showed no expression in the any organ of ‘Chiifu’ and ‘RcBr’. *BraACTIN* is used as control.

We selected 13 *BrIAA* genes to check their expression in different organs leaf, bud, flower, and siliqua between the parental lines Chiifu and RcBr using semi-quantitative RT-PCR. Primers for all 13 genes were designed by primer3 software and listed in S1 Table. The observed results demonstrated that *BrIAA* genes have similar and different expression among different organs as well as between Chiifu and RcBr. Moreover, BrIAA32 gene did not express in any of the selected organs from Chiifu and RcBr, showing the plausible nature of BrIAA32 as a pseudogene or its expression specificity in other organ(s) (Fig 5).

### Mutaions in *BrIAA* genes between *B*. *rapa* inbred lines

*B*. *rapa* Chiifu and RcBr inbred lines are morphologically different and facilitates to study the variations in genome as well as genes wide. The whole genome of RcBr was sequenced by illumina sequencer achieving 27x genome coverage after trimming low quality reads (data is unpublished). Draft genome *B*. *rapa* Chiifu line [8] was utilized as a reference to assemble the re-sequenced RcBr reads using bowtie software and possible SNPs were called by SAM and BCF tools (unpublished). In maximum 17 and 16 SNPs were identified from 5 and 3 *BrIAA* genes from A09 and A03 chromosome of *B*. *rapa*, respectively. There were more than 10 SNPs found in *BrIAA* genes of A01, A03, A05 and A09 chromosome. However, there were no SNP found in *BrIAA*12 and *BrIAA*27 genes of A07 chromosome. The *BrIAA*16 gene from Scaffold000203 which chromosome is unknown has two SNPs. While comparing SNPs block wise we found 24 SNPs from 10 genes of F block and, 16 and 15 SNPs from 2 and 7 genes of U and A blocks as maximum. But there is no SNP from *BrIAA*12 and *BrIAA*41 which are the only one *BrIAA* gene from block E and V (S2 Table).

## Discussion

Indoleacetic acid (IAA) is a plant growth hormone that regulates various plant growth and developmental genes expression [1, 15]. Isolation of Aux/IAA gene families in plants supports to understand their function in metabolic and developmental process [16, 17]. They are believed to play a vital role in early signaling [16]. Studies state that functions of Aux/IAA genes mainly depend on the expression pattern of the genes as effectively mutated Aux/IAA show expression changes [16]. There are numerous computational methods to identify gene families [10, 16, 18], however genome-wide searches are more effective in such identification. Hence we followed a genome wide search and identified 55 *B*. *rapa* Aux/IAA genes by HMM method. Reports on Aux/IAA genes have been available earlier, however few genes reported in their study as Aux/IAA genes did not contain the Aux/IAA domain hence they were not considered as members of Aux/IAA gene family [9]. Insilico mapping of genes in genome showed that 54 *BrIAA* genes were present in all 10 chromosomes of *B*. *rapa* while *BrIAA*16 was mapped in scaffold. These number of identified *BrIAA* genes were higher than Arabidopsis (30), rice (31), populus (35), and maize (31) despite their genome size.

In general *A*. *thaliana* genes are duplicated to multiple copies (in average three) in *B*. *rapa* genome [9], likely we found 54 *BrIAA* genes have higher homology with 30 *A*. *thaliana* genes. *BrIAA*32 is highly similar to *A*. *thaliana* ARF (AT5G60450) gene, however it was conserved with only one domain-IV. Studying the synteny and evolution of gene family becomes necessary after identification since *B*. *rapa* whole genome was triplicated between 13 and 17 million years ago with the complex history. The ancestral karyotype genome building blocks were decided for each *BrIAA* gene based on their *A*. *thaliana* homologs as well as by previously reported marker information [19]. The blocks A and F attained large number of *BrIAA* genes than any other blocks containing IAA genes. The F block was duplicated in A01, A03 and A05 chromosomes with same number of gene clusters except in A01 which missed one gene copy while it corresponds to *BrIAA*13 in A03 and *BrIAA*14 in A05. Few other gene clusters were observed from block A in A09 and A10 indicating the different number of complex segmental duplication event in *B*. *rapa* genome (Fig 1). Synteny analysis between *BrIAA* represented 16 segmental duplicated genes, among them 10 and 6 were having 2 to 3 copies in different chromosomes, whereas, 16 *BrIAA* genes were unique among all *B*. *rapa* genes (S3 Table). Interestingly block A possessed two tandem repeats in their duplicated regions in A09 and A10 while block F contained only one tandem repeat in A03 even though there were three duplicated IAA gene clusters in *B*. *rapa* which confers their importance in genome duplication.

Further evolutionary analysis of *BrIAA* genes with *A*. *thaliana*, *O*. *sativa* and *Z*. *mays* species elucidated the conservation and divergence of IAA genes among mono- and dicotyledonous plants. The constructed phylogenetic tree showed 3 major groups A, B and C which later divided into 11 subgroups (Fig 2). The subgroups A1, A2, A3 and B2 were conserved with IAA genes of all four species which denotes that 83 genes of these groups were originated before the divergence of monocots and dicots. However, we could observe 30 IAA genes from only dicot plants *B*. *rapa* and *A*. *thaliana*, whereas, 31 IAA genes from only monocot plants *O*. *sativa* and *Z*. *mays*. These results imply that these genes were either evolved or lost after monocot and dicotyledon divergence. Moreover, these genes confer their vital role in the development of plants of both mono and dicot species. Interestingly group C were completely rooted from mother clade evidencing the complete loss of group C genes in dicot crops and their importance in monocot specific traits. Further insights of phylogeny indicated that there were 23 sister pairs between *BrIAA* and *AtIAA* gene, whereas, no single *AtIAA-AtIAA* sister pair was observed which could represent the genome conservation between *A*. *thaliana* and *B*. *rapa*. Moreover, there were 6 *BrIAA*-*BrIAA* pairs found since this ‘mesohexaploidy’ plant *B*. *rapa* had triplicated genome. Monocotyledon plant IAA genes were found with 13 *OsIAA-ZmIAA* sister pairs while 2 and 7 numbers of *OsIAA-OsIAA* and ZmIAA-ZmIAA sister pairs indicated the evolutionary relationship among them. Overall, no sister pair was observed between mono- and dicot species in phylogentic tree which could indicate the divergence and conservation of IAA genes among these four species.

Investigation of 55 *BrIAA* proteins using MEME tool revealed the changes in conserved domain and motif architecture, accordingly 37 *BrIAA* proteins were conserved with all four domains, however, eight proteins (*BrIAA*30, *BrIAA*31, *BrIAA*32, *BrIAA*38, *BrIAA*42, *BrIAA*46, *BrIAA*51 and *BrIAA*55) were lacking domain-I and -II (Fig 2, S3 Fig & S4 Fig). Domain-I is a repression domain and domain II interacts with the F-box protein TIR1 [4]. Moreover, mutations or absence of these domains could prolong life of these proteins compared to other standard Aux/IAA proteins [8]. Their counterpart proteins from *A*. *thaliana*’s sister pair also lacking either one or both the domain-I and -II suggesting their evolutionarily conservation in both the species. As previous studies on *A*. *thaliana*, these 8 genes of *B*. *rapa* may insensitive to IAA treatment since they have longer half-lives [2].

Expression profiles of Aux/IAA genes were studied by comparing with ESTs, microarray and conducting semi qRT-PCR analysis for selected genes since functions of the Aux/IAA genes depends on the expression of the genes [15]. Comparative analysis of *BrIAA* genes with ESTs and microarray data showed that there were no any similarity found for seven genes (*BrIAA26*, *BrIAA27*, *BrIAA30*, *BrIAA31*, *BrIAA32*, *BrIAA52*, and BrIAA55) those missing one or more than one domain (Table 1, Table 2 & S3 Fig). Among the seven genes *BrIAA30* and *BrIAA32* were conserved with only domain-IV and *BrIAA31* with only domain-III. Semi qRT-PCR analysis evidenced *BrIAA30* and *BrIAA31* has very low expression in leafs of Chiifu and RcBr, and no expression in Chiifu siliqua whereas *BrIAA32* showed no expression in any experimented organs(S5 Fig). In our study many different *BrIAA* genes showed similar expression pattern which might possibly change expression level after diverse stress treatments like in other plants [13, 14]. Eventhough Chiifu and RcBr lines having different phenotypes and growth cycles, showed a similar expression pattern for many of the genes. Conversing in the case of *BrIAA51*, a transcription regulator was highly expressive in bud, leaf and flower of RcBr than Chiifu (Table 2, Fig 4 & S2 Fig). Interestingly two sister pairs (*BrIAA24* and *BrIAA25*, *BrIAA33* and *BrIAA53*) were observed with similar expression pattern in both the lines, suggesting that they may perform redundant functions. Another sister pair from semi qRT-PCR result (*BrIAA39* and *BrIAA40*) showed significant expression difference in flower and siliqua indicates that these genes had functional divergence after duplication event. There was no SNP found in *BrIAA39* and *BrIAA40* genes even though they have considerable expression variation between Chiifu and RcBr. Similarly *BrIAA32* which is highly similar to ARF gene family in *A*. *thaliana* has no SNP on full length gene sequence and showed no expression in both the lines which proposes this gene might be organ specific or silenced by some promoter or transcription factor. There is wide expression difference of *BrIAA33* in siliqua and mature leaf of Chiifu and RcBr (Fig 4, & Table 2), and they found with three SNPs on exon and two on intronic region. The gain-of-functional mutation in *BrIAA33* gene reducing the response to auxin and suppresses the fertility and plant growth which was reported in A. thaliana [18]. The *BrIAA51* gene was highly involved in non-host resistance (NHO1) with bacterial virulence and also it’s responses to cell-specific nitrogen mediate development in plants [20, 21]. Even though SNPs in this gene were located in only intronic regions, we found two Indels (-ATGA and +AT) in the two exonic region of RcBr, while the gene showed high expression in bud, flower and siliqua of RcBr and minimal in Chiifu (Fig 4).

We have selected few genes in *B*. *rapa* and have analyzed their expression pattern based on IAA treatment. The expression of BrIAA gene was higher than the control upon IAA treatment at various exposure times. From the present study, we conclude that exposure time played an important role in up-regulation/down-regulation of aux gene upon IAA treatment. Expression pattern of gene in specific organs at a given exposure time is the significant requirement to subsequent elucidation of corresponding protein required for proper execution of developmental, metabolic and signalling processes [13]. The mRNA levels of aux genes *BrIAA1*, *BrIAA6*, *BrIAA14*, *BrIAA15*, *BrIAA32* and *BrIAA33* were maximum at 48 h exposure time suggesting that these five aux genes might play an essential role in the development of leaf. The difference in expression levels of Aux/IAA genes is likely due to a variety of factors such as tissue specific auxin reception, cell-type dependence, differential regulation of free auxin concentrations, different modes of auxin-dependent transcriptional and posttranscriptional regulation [22]. It has been reported earlier that the diversity of numbers and locations of auxin signaling transduction-related cis-elements may partially account for the difference in expression patterns of IAAs under IAA treatment in Tomato [23]. Aux/IAA family genes of rice have responded to exogenous IAA in a highly differential fashion with respect to dosage and time [22]. Likewise, the exogenous treatment of IAA had presented differential transcript levels of Aux/IAA genes in Arabidopsis, maize, and tomato [24, 25, 26]. Besides their association in growth and development the Aux/IAA genes also involved in stress/defense signalling in rice [27, 28]. When we analyzed the involvement of BrIAA to abiotic stress we found that 21 of the 55 candidate genes were differently expressed. Alike sorghum, we also observed that IAA (BrIAA) genes in *B*. *rapa* could be involved in abiotic stress response [28]. The genes *BrIAA3*, *BrIAA13*, *BrIAA15*, *BrIAA23*, *BrIAA43*, *BrIAA48* and *BrIAA54* were found to differently express during all the treatments marking their involvement in abiotic stress response.

## Conclusion

A genome wide analysis and characterization of Aux/IAA family genes in *B*. *rapa* was carried out in the current study. The Aux/IAA gene family was densely populated with 55 genes in *B*. *rapa* than the other plants like arabidopsis, rice, populus and maize. The identified genes were characterized based on phylogeny; motif pattern, expression analysis and their stress response have also been investigated. We found the genes are well conserved among *A*. *thaliana*, *O*. *sativa* and *Z*. *mays*, however, variations were observed in the conservation and divergence of IAA genes among mono- and dicotyledonous plants. Expression analysis revealed the involvement of the *BrIAA* genes in plant development such as leaf growth and flower maturation. Alike other plants, the *BrIAA* family genes were also affected by different stress treatments. Seven genes were analysed for their reponse to Auxin treatment. All the genes were upregulated to lower auxin treatment and down regulated by higher treatment. Among the 55 genes identified, 21 genes were differently expressed to cold, drought and salt stress treatments, especially, the genes *BrIAA*3, *BrIAA*13, *BrIAA*15, *BrIAA*23, *BrIAA*43, *BrIAA*48 and *BrIAA*54 were highly responsive to all three. Identification of SNPs between Chiifu and RcBr are evidencing that functional mutation in IAA genes may play key role in the plant growth cycle. We believe our data would be a major resource for further studies on functional genomics of Aux/IAA genes and open the opportunities in plant genomics and crop breeding.

## Supporting Information

S1 Fig Five conserved motifs of 144 *Aux/IAA* proteins from *A*. *thaliana*, *B*. *rapa*, *O*. *sativa* and *Z*. *mays*. Number of sites corresponds to the number of motif occurrences in 144 proteins. Each Amino acid height in motif logo represents the conservation in the total number of sites.
(TIF)

S2 Fig Biological process and molecular function of *BrIAA* genes. Filtering cutoff was set to 5 in level-2annotation.
(PDF)

S3 Fig Multiple sequence alignment of 55 *BrIAA* protein sequences. Five motifs and four domains are highlighted by red color lines under the amino acid position. Domain IV consist of two motifs, namely, motif 1and 5 in motif logo.
(PDF)

S4 Fig Phylogenetics analysis and motif distribution of 55 *BrIAA* proteins. Phylogenetics tree was constructed by Neighbor-joining method with 1000 bootstrap replications and values were given on each node. Motifs for each protein were predicted by MEME web server and motif height in each gene symbolizes the conservation.
(TIF)

S5 Fig Semi qRT-PCR analysis of *BrIAA30* and *BrIAA31* in the different organs of ‘Chiifu’ and ‘RcBr’. (TIF)

S1 Table Primer information used for the semi qRT-PCR. (XLSX)

S2 Table Single Nucleotide Polymorphisms identified between Chiifu and RcBr lines along with the strand and block information. (XLSX)

S3 Table Synteny analysis between the *BrIAA* genes. (XLSX)
