# Supplementary figures and images for: Genome-Wide Analysis and Characterization of Aux/IAA Family Genes in Brassica rapa
Source: PLoS One. 2016 Apr 6;11(4):e0151522. doi: 10.1371/journal.pone.0151522 (PMC4822780; doi:10.1371/journal.pone.0151522)

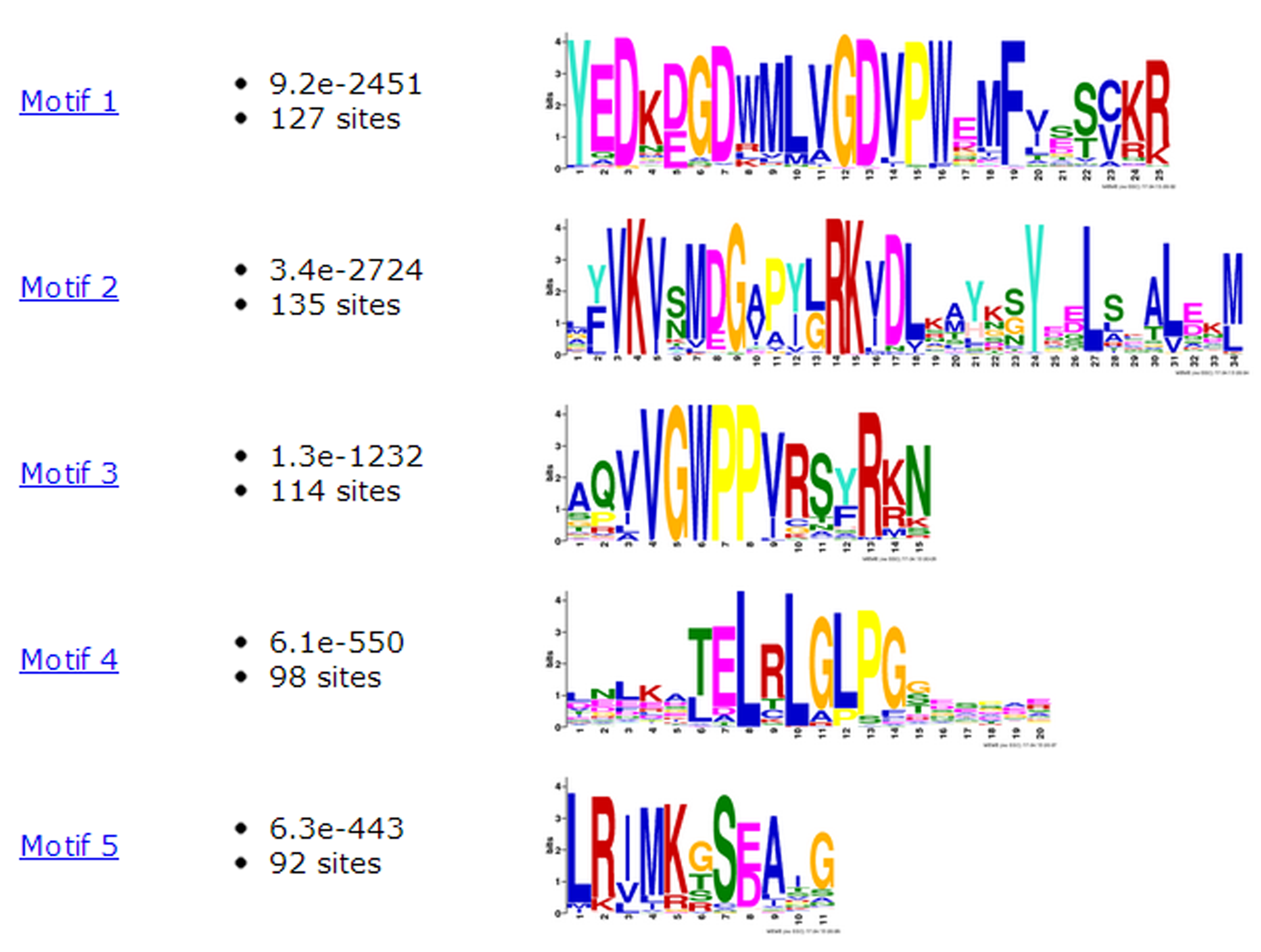

Supplement: S1 Fig — Number of sites corresponds to the number of motif occurrences in 144 proteins. Each Amino acid height in motif logo represents the conservation in the total number of sites. (TIF) [file pone.0151522.s001.tif]

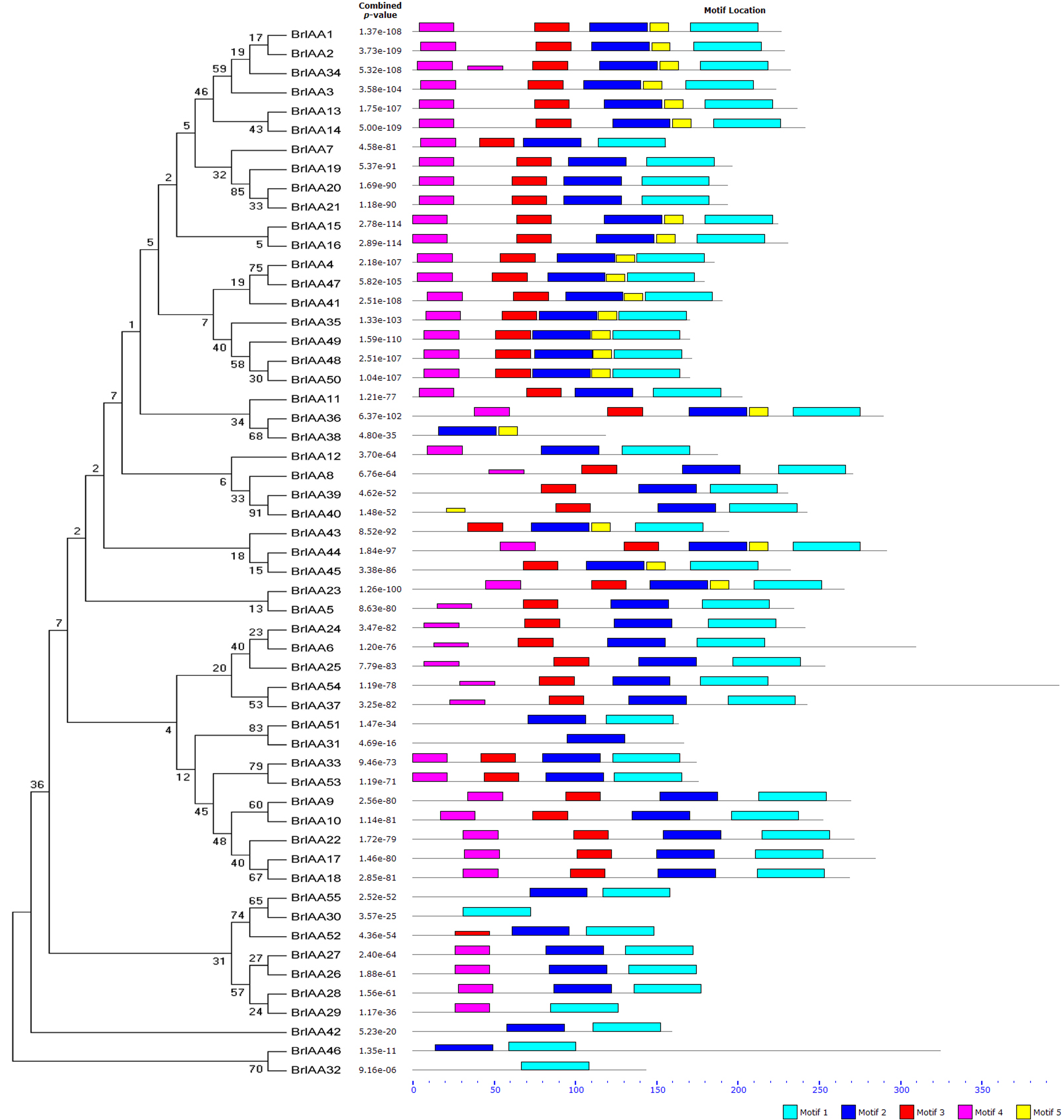

Supplement: S4 Fig — Phylogenetics tree was constructed by Neighbor-joining method with 1000 bootstrap replications and values were given on each node. Motifs for each protein were predicted by MEME web server and motif height in each gene symbolizes the conservation. (TIF) [file pone.0151522.s004.tif]

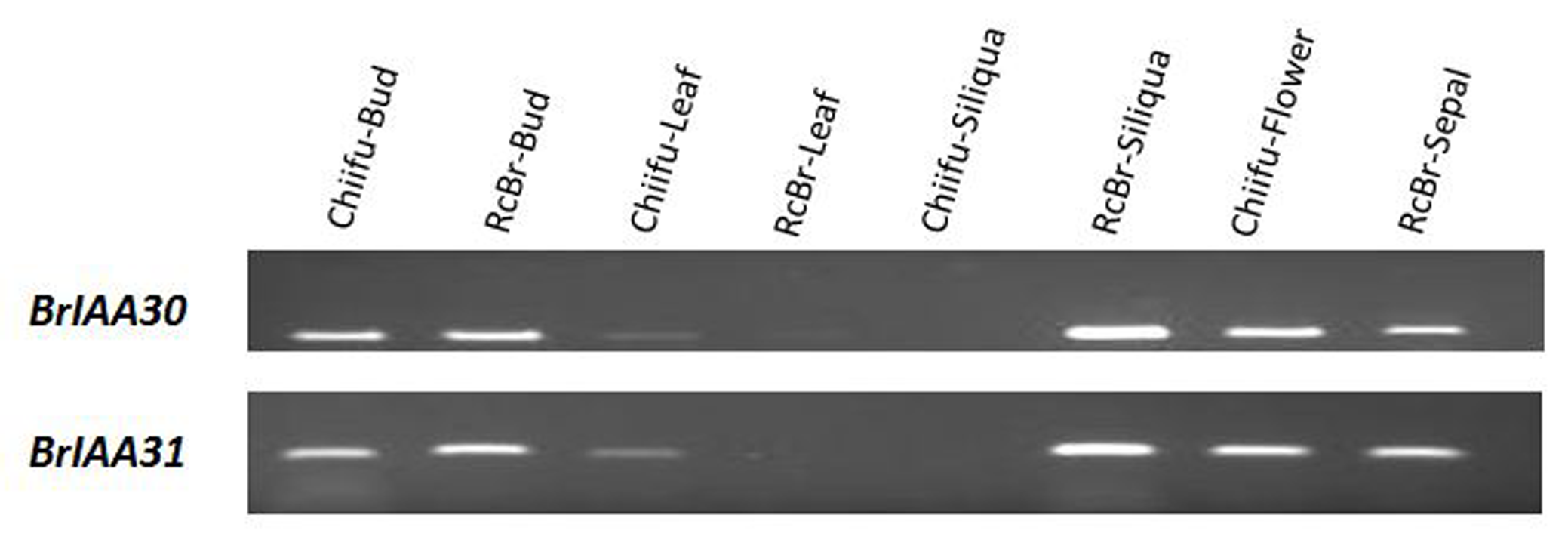

Supplement: S5 Fig — (TIF) [file pone.0151522.s005.tif]
