# Supplementary material for: Genome-Wide Analysis and Characterization of Aux/IAA Family Genes in Brassica rapa
Source: PLoS One. 2016 Apr 6;11(4):e0151522. doi: 10.1371/journal.pone.0151522 (PMC4822780; doi:10.1371/journal.pone.0151522)
Supplement: S2 Fig — Filtering cutoff was set to 5 in level-2annotation. (PDF) [file pone.0151522.s002.pdf]

# Sequence distribution: biological_process(Filtered by #Seqs: cutoff=5.0)

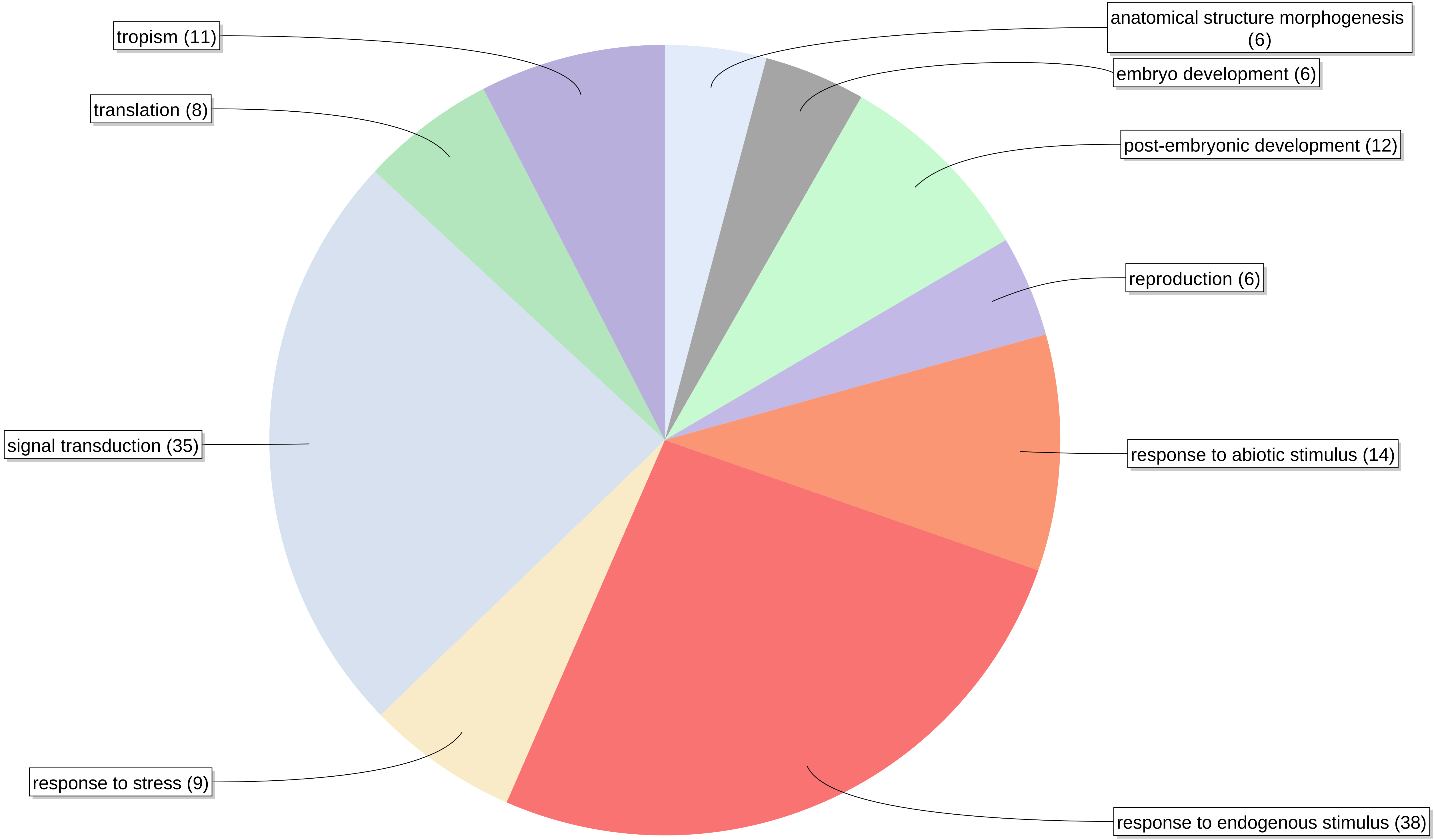

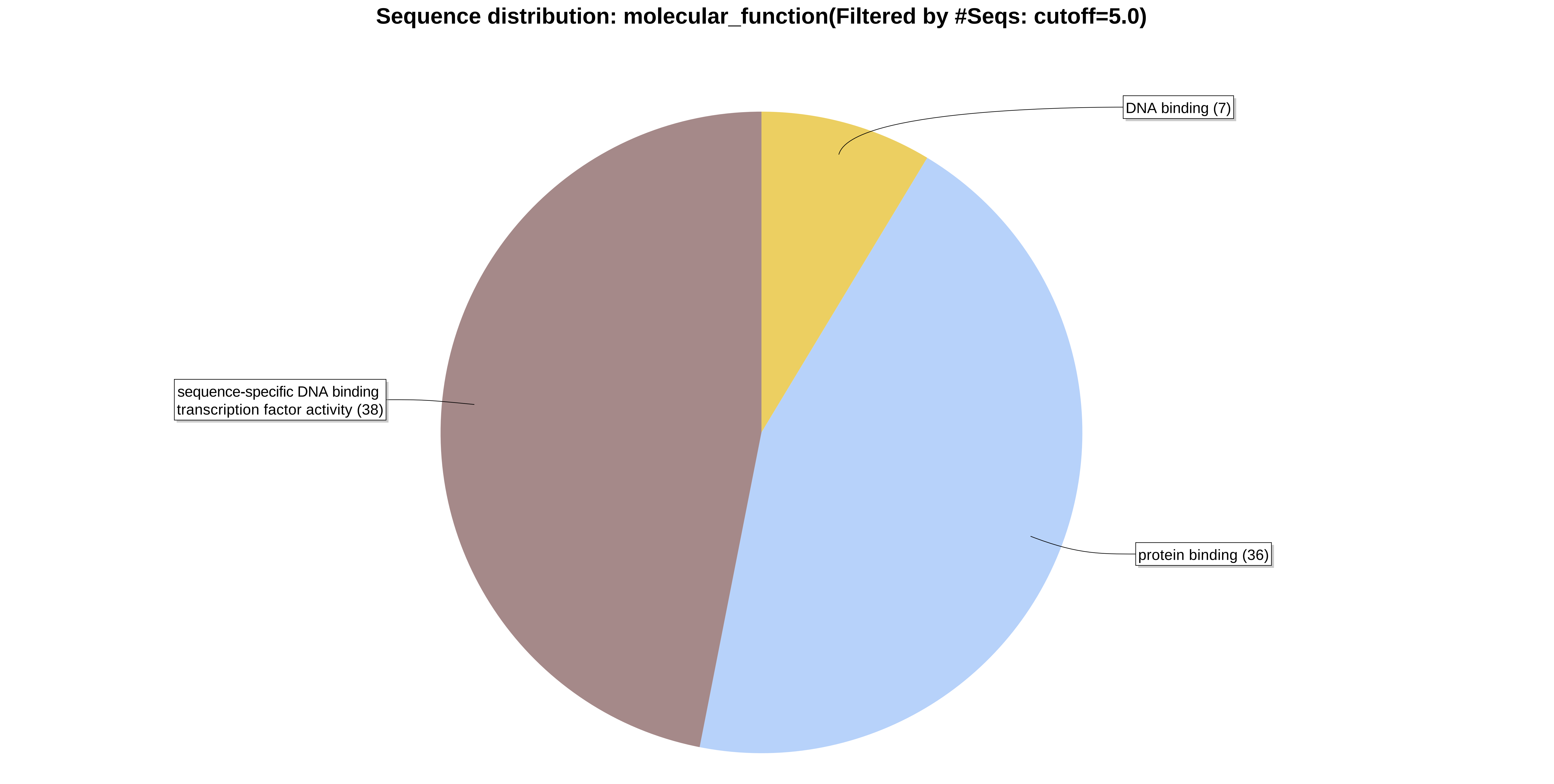
Sequence distribution: molecular_function(Filtered by #Seqs: cutoff=5.0)
DNA binding (7)
sequence-specific DNA binding
transcription factor activity (38)
protein binding (36)
