# Supplementary material for: Genome-Wide Analysis and Characterization of Aux/IAA Family Genes in Brassica rapa
Source: PLoS One. 2016 Apr 6;11(4):e0151522. doi: 10.1371/journal.pone.0151522 (PMC4822780; doi:10.1371/journal.pone.0151522)
Supplement: S3 Fig — Five motifs and four domains are highlighted by red color lines under the amino acid position. Domain IV consist of two motifs, namely, motif 1and 5 in motif logo. (PDF) [file pone.0151522.s003.pdf]

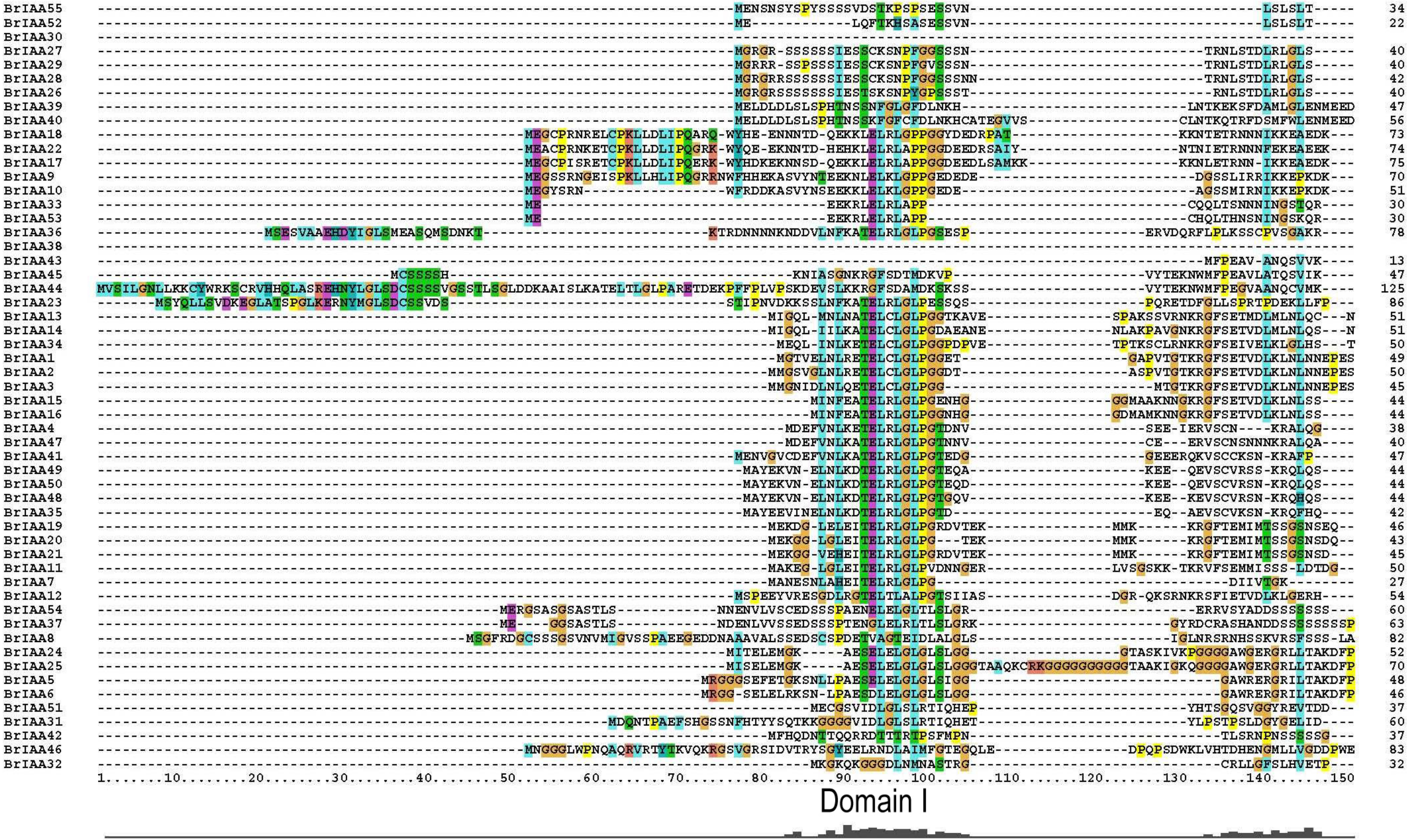
BrIAA55
BrIAA52
BrIAA30
BrIAA27
BrIAA29
BrIAA28
BrIAA26
BrIAA39
BrIAA40
BrIAA18
BrIAA22
BrIAA17
BrIAA9
BrIAA10
BrIAA33
BrIAA53
BrIAA36
BrIAA38
BrIAA43
BrIAA45
BrIAA44
BrIAA23
BrIAA13
BrIAA14
BrIAA34
BrIAA1
BrIAA2
BrIAA3
BrIAA15
BrIAA16
BrIAA4
BrIAA47
BrIAA41
BrIAA49
BrIAA50
BrIAA48
BrIAA35
BrIAA19
BrIAA20
BrIAA21
BrIAA11
BrIAA7
BrIAA12
BrIAA54
BrIAA37
BrIAA8
BrIAA24
BrIAA25
BrIAA5
BrIAA6
BrIAA51
BrIAA31
BrIAA42
BrIAA46
BrIAA32
Domain I

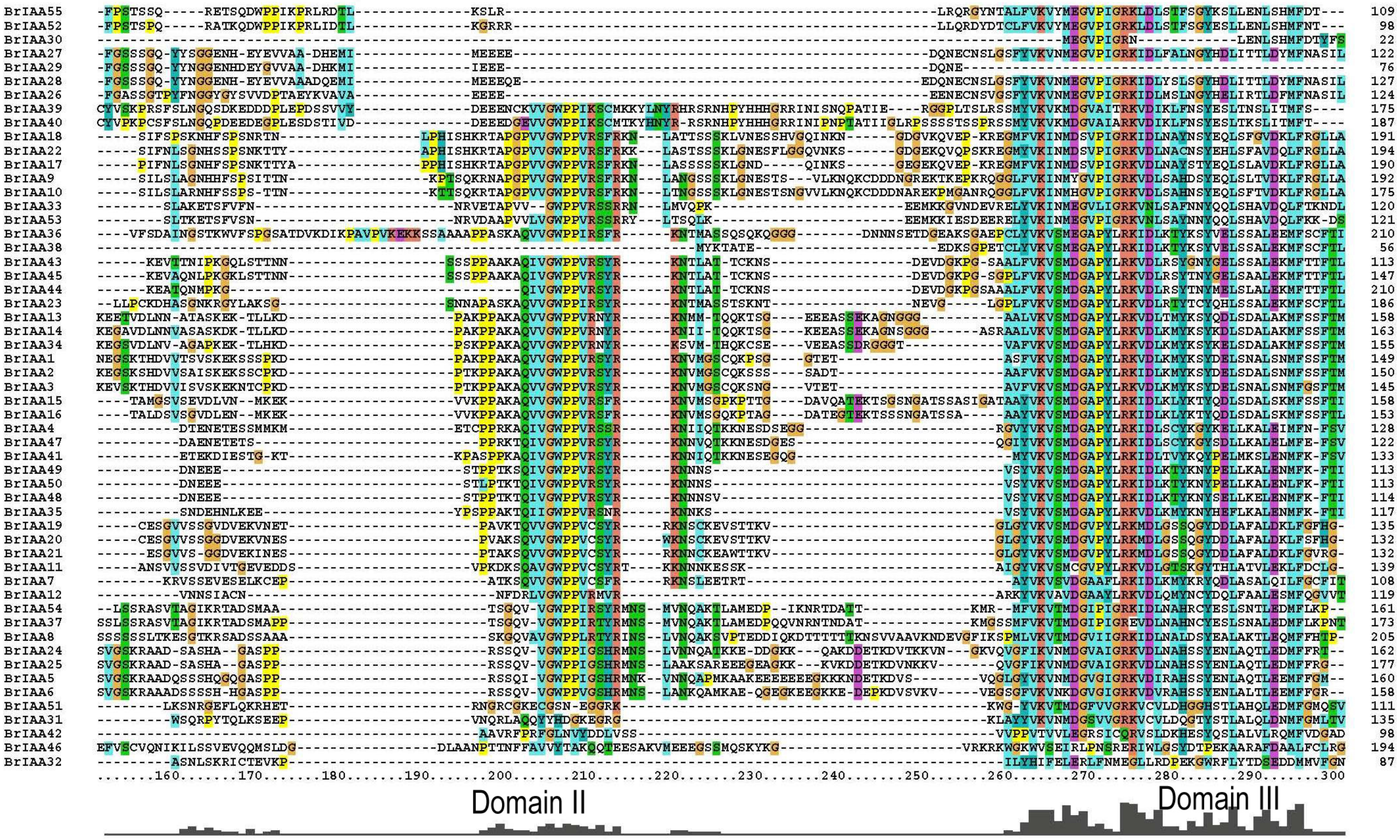
Domain II
Domain III

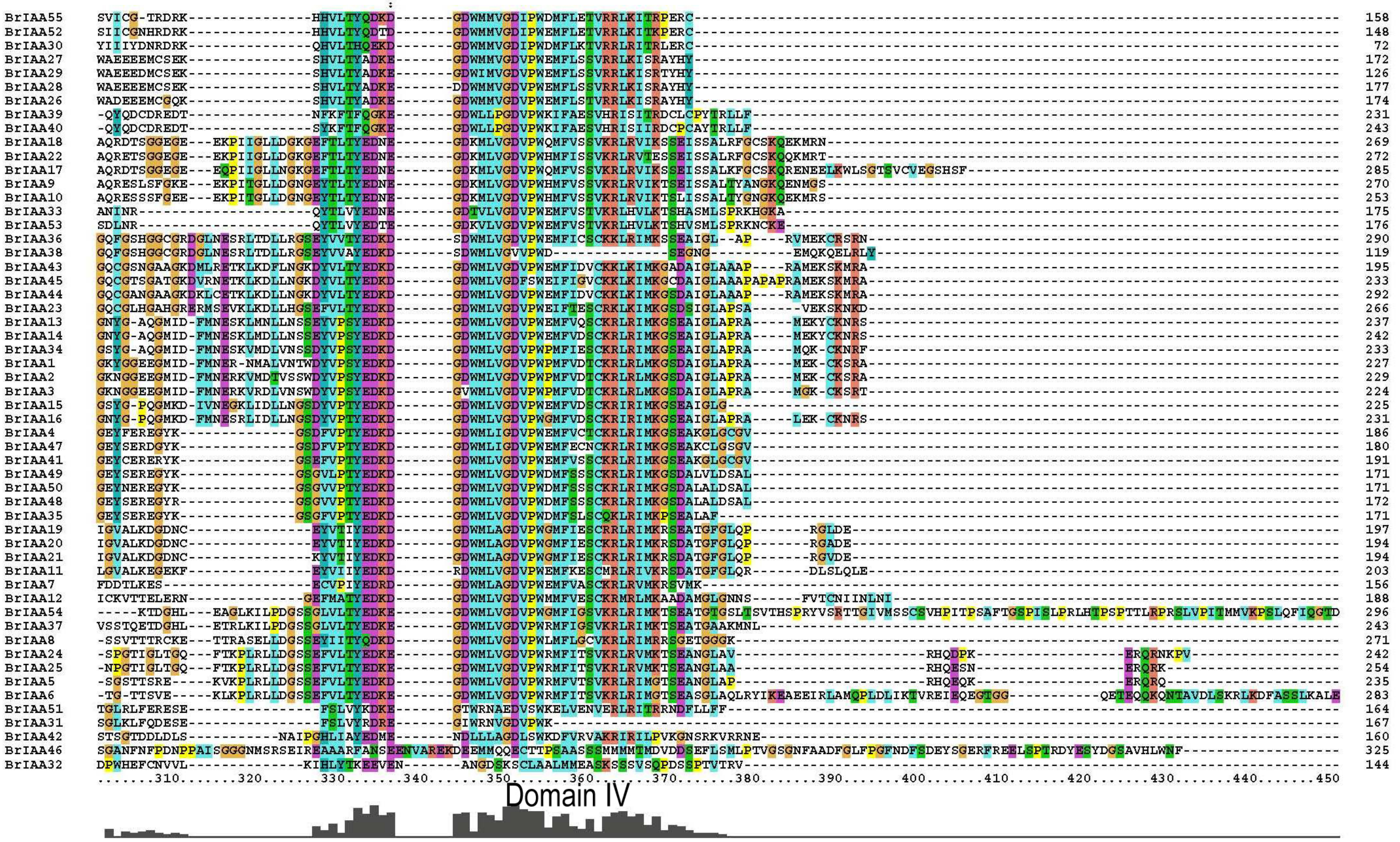
BrIAA55 158
BrIAA52 148
BrIAA30 72
BrIAA27 172
BrIAA29 126
BrIAA28 177
BrIAA26 174
BrIAA39 231
BrIAA40 243
BrIAA18 269
BrIAA22 272
BrIAA17 285
BrIAA9 270
BrIAA10 253
BrIAA33 175
BrIAA53 176
BrIAA36 290
BrIAA38 119
BrIAA43 195
BrIAA45 233
BrIAA44 292
BrIAA23 266
BrIAA13 237
BrIAA14 242
BrIAA34 233
BrIAA1 227
BrIAA2 229
BrIAA3 224
BrIAA15 225
BrIAA16 231
BrIAA4 186
BrIAA47 180
BrIAA41 191
BrIAA49 171
BrIAA50 171
BrIAA48 172
BrIAA35 171
BrIAA19 197
BrIAA20 194
BrIAA21 194
BrIAA11 203
BrIAA7 156
BrIAA12 188
BrIAA54 296
BrIAA37 243
BrIAA8 271
BrIAA24 242
BrIAA25 254
BrIAA5 235
BrIAA6 283
BrIAA51 164
BrIAA31 167
BrIAA42 160
BrIAA46 325
BrIAA32 144
310 320 330 340 350 360 370 380 390 400 410 420 430 440 450
Domain IV
